# Advancing Ki67 hotspot detection in breast cancer: a comparative analysis of automated digital image analysis algorithms

**DOI:** 10.1111/his.15294

**Published:** 2024-08-05

**Authors:** Mieke C Zwager, Shibo Yu, Henk J Buikema, Geertruida H de Bock, Thomas W Ramsing, Jeppe Thagaard, Timco Koopman, Bert van der Vegt

**Affiliations:** ^1^ Department of Pathology University of Groningen, University Medical Center Groningen Groningen The Netherlands; ^2^ Department of Epidemiology University of Groningen, University Medical Center Groningen Groningen The Netherlands; ^3^ Visiopharm Hørsholm Denmark; ^4^ Pathologie Friesland Leeuwarden The Netherlands

**Keywords:** artificial intelligence (AI), breast cancer, digital image analysis (DIA), hotspot, Ki67 proliferation index

## Abstract

**Aim:**

Manual detection and scoring of Ki67 hotspots is difficult and prone to variability, limiting its clinical utility. Automated hotspot detection and scoring by digital image analysis (DIA) could improve the assessment of the Ki67 hotspot proliferation index (PI). This study compared the clinical performance of Ki67 hotspot detection and scoring DIA algorithms based on virtual dual staining (VDS) and deep learning (DL) with manual Ki67 hotspot PI assessment.

**Methods:**

Tissue sections of 135 consecutive invasive breast carcinomas were immunohistochemically stained for Ki67. Two DIA algorithms, based on VDS and DL, automatically determined the Ki67 hotspot PI. For manual assessment; two independent observers detected hotspots and calculated scores using a validated scoring protocol.

**Results:**

Automated hotspot detection and assessment by VDS and DL could be performed in 73% and 100% of the cases, respectively. Automated hotspot detection by VDS and DL led to higher Ki67 hotspot PIs (mean 39.6% and 38.3%, respectively) compared to manual consensus Ki67 PIs (mean 28.8%). Comparing manual consensus Ki67 PIs with VDS Ki67 PIs revealed substantial correlation (*r* = 0.90), while manual consensus versus DL Ki67 PIs demonstrated high correlation (*r* = 0.95).

**Conclusion:**

Automated Ki67 hotspot detection and analysis correlated strongly with manual Ki67 assessment and provided higher PIs compared to manual assessment. The DL‐based algorithm outperformed the VDS‐based algorithm in clinical applicability, because it did not depend on virtual alignment of slides and correlated stronger with manual scores. Use of a DL‐based algorithm may allow clearer Ki67 PI cutoff values, thereby improving the clinical usability of Ki67.

## Graphical abstract

Manual assessment of Ki67 hotspots is difficult and prone to variability. Automated Ki67 hotspot assessment correlated strongly with manual Ki67 assessment and provided higher Ki67 PIs. DL‐based automated Ki67 assessment has high clinical applicability, because it does not depend on virtual alignment of slides and correlates strongly with manual scoring.

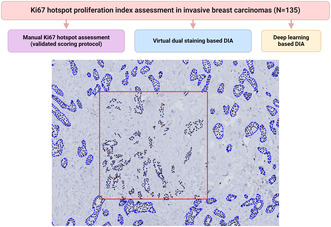

**Abbreviations**
DIA: digital image analysis
DL: deep learning
ER: oestrogen receptor
H&E: haematoxylin and eosin
HER2: human epidermal growth factor receptor 2
ICC: intraclass correlation coefficient
IKWG: International Ki67 in Breast Cancer Working Group
NST: no special type
PI: proliferation index
PR: progesterone receptor
ROI: region of interest
UMCG: University Medical Center Groningen
VDS: virtual dual staining

## Introduction

Breast cancer is one of the most common malignancies and the leading cause of cancer‐related death in women worldwide.^1^, ^2^ The Ki67 proliferation index (PI) is widely used as a prognostic and predictive marker in breast cancer.^3^ Ki67 is a nuclear protein that is present during all phases of the cell cycle but is not expressed by cells in G0.^4^, ^5^, ^6^ It is therefore expressed in all proliferating cells and can be used as an indicator of tumour proliferation.^3^, ^7^

Ki67 is prone to intratumoural heterogeneity and a general lack in standardized and reproducible counting methods has limited the use of the Ki67 PI in clinical practice.^6^, ^8^, ^9^, ^10^ Digital image analysis (DIA) algorithms provide an efficient and reproducible alternative to manual analysis of Ki67 in breast cancer.^11^, ^12^, ^13^, ^14^, ^15^, ^16^, ^17^ In a previous study, we have shown that DIA is an accurate method for assessing the Ki67 PI on whole slides of invasive breast carcinomas in the routine diagnostic setting.^12^ The high intermodality agreement between manual assessment and DIA confirmed that DIA offers an accurate, objective alternative to manual assessment.

It is assumed that a hotspot is a biologically active part of the tumour and is therefore relevant for disease outcome.^11^ Several studies have shown that Ki67 hotspot scoring is equal to or better than global whole‐slide scoring in predicting patient outcome.^18^, ^19^, ^20^, ^21^ However, visual detection of Ki67 hotspots is difficult and manual scoring is labour‐intensive, time‐consuming, and prone to inter‐ and intraobserver variability.^6^, ^10^ Although a standardized protocol for the manual scoring of Ki67 hotspots has been proposed in order to decrease this variability, interobserver reproducibility remains suboptimal compared to global whole‐slide scoring, making this method unsuitable for use in clinical practice.^22^

While it has been shown that DIA can outperform manual assessment of Ki67 in both whole‐tumour sections and in manually predefined hotspots, visual detection of a hotspot in a tumour is still a challenge.^11^ Recently DIA algorithms, including deep learning (DL)‐driven algorithms, that are able to both detect and score Ki67 hotspots have emerged. The aim of this study was to compare the clinical performance of Ki67 hotspot detection and scoring DIA algorithms based on virtual dual staining (VDS) and DL with manual Ki67 hotspot PI assessment.

## Materials and Methods

### Patient selection

Resection specimens of 154 consecutive primary invasive breast carcinomas diagnosed at the University Medical Center Groningen (UMCG, The Netherlands) between August 2015 and February 2017 were included retrospectively. Nineteen cases were excluded due to immunohistochemical or image data errors prior to the start of the analysis. Therefore, 135 cases were available for analysis. Patient and tumour characteristics are shown in Table 1. Material used in this study was obtained from diagnostic archival material stored at the Department of Pathology. This retrospective study was deemed to fall outside the scope of the Medical Research Involving Human Subjects Act (WMO) by the Ethical Committee of the UMCG (ValiDIApath, research register number 16818, UMCG research register number 18848); therefore, informed consent was not required according to the Dutch Law for Medical Research and institutional guidelines. No objection to research on redundant diagnostic material was recorded from these patients in the institutional record of objection. All patient material was handled according to the ‘Code of conduct for health research’ of the Dutch Federation of Biomedical Scientific Societies.^23^

**Table 1:** Patient and tumour characteristics

	All cases, *n* (%)
Total	135 (100)
Gender	
Female	132 (97.8)
Male	3 (2.2)
Age (years)	
<60	63 (46.7)
≥60	72 (53.3)
Mean	60.4 years
Histological type	
Invasive carcinoma NST	114 (84.4)
Invasive lobular carcinoma	18 (13.3)
Mixed invasive carcinoma NST/invasive lobular carcinoma	3 (2.2)
Histological grade	
1	28 (20.7)
2	67 (49.6)
3	40 (29.6)
Tumour diameter (cm)	
≤2	89 (65.9)
>2 and ≤5	38 (28.1)
>5	8 (5.9)
Mean	2.8 cm
ER	
Positive	114 (84.4)
Negative	21 (15.6)
PR	
Positive	100 (74.1)
Negative	35 (25.9)
HER2	
Positive	13 (9.6)
Negative	122 (90.4)

ER, oestrogen receptor; HER2 human epidermal growth factor receptor 2; NST, no special type; PR, progesterone receptor.

### Immunohistochemistry

Serial sections of 3 μm were cut from the formalin‐fixed paraffin‐embedded tumour blocks in the routine diagnostic process. Sections were immunohistochemically stained for Ki67 (30‐9, rabbit monoclonal antibody, Ventana Roche, Oro Valley, AZ, USA) and CK8/18 (B22.1 & B23.1, mouse monoclonal antibody, Ventana Roche). The antibodies were prediluted by the manufacturer and sections were stained on a Ventana Benchmark Ultra immunostainer (Ventana) according to the manufacturer's protocols.

### Manual hotspot scoring

Digital images were obtained using a Philips Ultra Fast Scanner (Philips, Best, Netherlands).

Manual scoring of the Ki67 PI was performed by two observers (M.Z. and S.Y.). Both observers independently performed visual detection of a hotspot on each tumour section image, defined as an invasive tumour area with the apparent highest proliferation rate, and scored Ki67 within the hotspot using a standardized scoring protocol for Ki67 immunohistochemistry on whole sections.^22^ Any intensity of brown nuclear staining was considered positive. A region of interest (ROI) was annotated within or around the hotspot. In each ROI, 500 tumour cells were counted in a ‘typewriter’ pattern. The number of Ki67‐positive cells was divided by the total number of cells counted to calculate the Ki67 hotspot PI.

### VDS‐based DIA

Automated hotspot detection and scoring using the VDS‐based algorithm on whole slides was performed in VIS version 2019.02 using the Hot Spot APP (20114—Hot spot, version 1.0, Visiopharm, Horsholm, Denmark). On the CK8/18 + Ki67 VDS image, tumour areas were outlined by the PCK VDS Tumour Detection algorithm. Noninvasive tumour areas and benign epithelial structures were manually discarded. Subsequently, Ki67 quantification within these areas was performed by detecting nuclei based on their shape and size and classifying them into either Ki67‐positive or Ki67‐negative based on the nuclear staining. A heatmap was created, and a hotspot was automatically identified based on the ratio between Ki67‐positive nuclei and the total number of nuclei in the invasive tumour regions. The shape and size of the hotspot was predefined as a square of 0.5 mm^2^ containing a minimum of 500 cells. Within this hotspot, Ki67 was quantified and the Ki67 hotspot PI was calculated by dividing the number of Ki67‐positive cells by the total number of cells counted in the hotspot.

### DL‐based DIA

**Figure 1:**
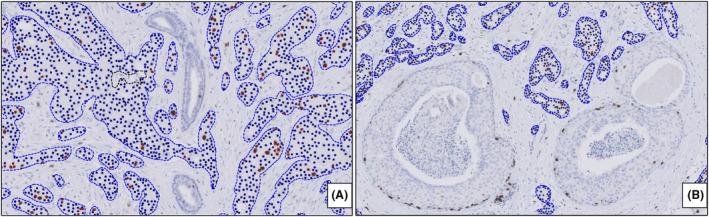
Tumour detection by the DL‐based algorithm. Benign epithelial structures (**A**) and carcinoma *in situ* (**B**) are automatically discarded by the algorithm. [Color figure can be viewed at wileyonlinelibrary.com]

Automated hotspot detection and scoring using the DL‐based algorithm was performed in VIS version 2019.02 using the Hot Spot APP (20114—Hot spot, version 1.0, Visiopharm). On the Ki67‐stained slide, tissue was detected by the Tissue Detect APP and invasive tumour areas were outlined by the Tumour Detection APP (10162—IHC, Tumour Detection, AI). In contrast to the VDS‐based DIA, noninvasive tumour areas and benign epithelial structures are automatically discarded by the AI Tumour Detection APP (Figure 1). Subsequently, Ki67 quantification was performed by detecting nuclear detection and classification into either Ki67‐positive or ‐negative based on the nuclear staining (10173—Ki67, Breast Cancer, AI). A heatmap was created, and a hotspot (square of 0.5 mm^2^ containing a minimum of 500 cells) was automatically identified based on the ratio between Ki67‐positive nuclei and total number of nuclei in the invasive tumour regions.

### Statistical analysis

Correlations between the manual Ki67 hotspot PIs of the two observers, and between manual Ki67 hotspot PIs and DIA using VDS and DL were assessed using Spearman correlation. Interobserver agreement was visualized with scatterplots and Bland–Altman plots. A paired samples *t*‐test was performed to compare means (Table S1). Analyses were performed in IBM SPSS statistics v. 25 (SPSS, Chicago, IL, USA).

## Results

**Figure 2:**
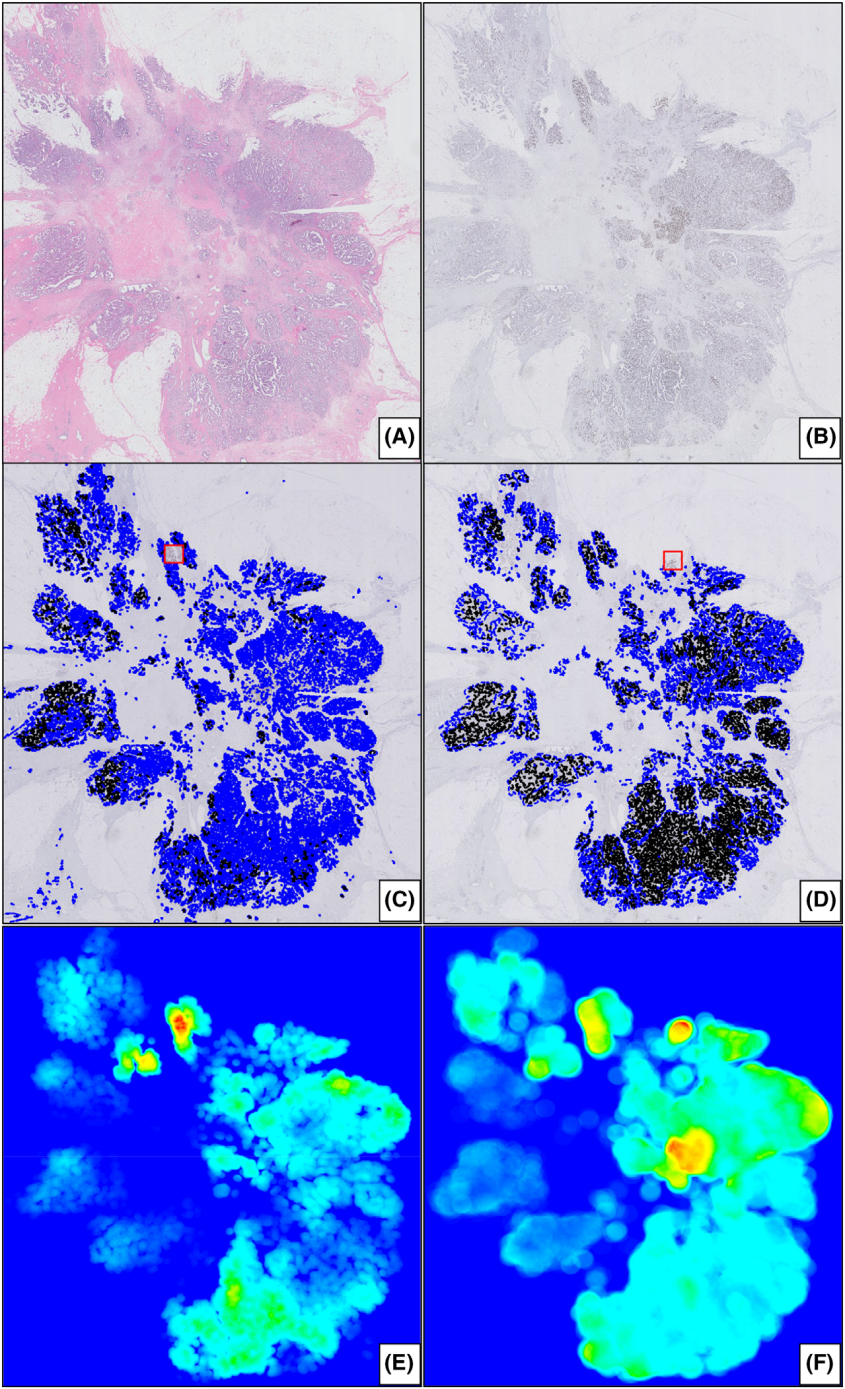
H&E‐stained slide (**A**), Ki67‐stained slide (**B**), results from the VDS‐based DIA (**C**), results from the DL‐based DIA (**D**), and the corresponding heatmaps (E and F, respectively). [Color figure can be viewed at wileyonlinelibrary.com]

Of the 135 cases available for analysis, in 37 (27%) cases VDS failed because of misalignment due to suboptimal quality of the sections or because sections were not correctly cut in serial order. Therefore, for the VDS, analyses were performed on 98 cases. Ki67 hotspot detection and scoring by using the DL‐based algorithm could be performed in all 135 cases. Figure 2 shows a representative example of a tumour stained for haematoxylin and eosin (H&E) and Ki67, the results of the VDS‐based DIA and DL‐based DIA, and the corresponding heatmaps.

### Comparison of manual observers

**Figure 3:**
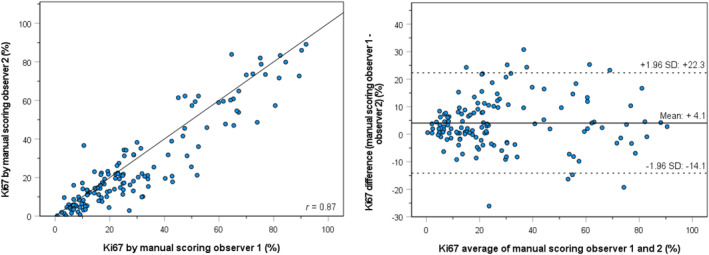
Scatterplot with the correlation coefficient (*r*) (left) and Bland–Altman plot of agreement (right) between the Ki67 hotspot PIs by manual scoring by the two observers. [Color figure can be viewed at wileyonlinelibrary.com]

The Ki67 hotspot PIs from both observers obtained by manual hotspot detection and scoring are displayed in Table 2. Ki67 hotspot PIs differed between the two observers, with a mean of 30.8% versus 26.7% and a median of 23.9% versus 19.3%. Figure 3 shows a scatterplot and Bland–Altman plot comparing the manual Ki67 hotspot PIs of the two observers. Interobserver agreement between the manual observers was high (*r* of 0.87). Given the high interobserver agreement, the manual scores were averaged and considered as the manual consensus Ki67 PIs (Table 2). These manual consensus PIs were used in the subsequent analyses. Results of the analyses with the individual manual scores can be found in Table S2.

**Table 2:** Ki67 hotspot proliferation indices (%) by manual scoring and digital automated hotspot detection analysis

	Mean	Min	Q1	Q2 (median)	Q3	Max
Cells counted						
Manual observer 1	503.9	500	501	503	506	516
Manual observer 2	513.4	500	507	512	519	533
VDS‐based DIA	912.9	540	638	700.5	1002.3	2907
DL‐based DIA	817.8	548	644	709	784	3105
Ki67 score (%)						
Manual observer 1	30.8	0.8	11.8	23.9	46.8	91.9
Manual observer 2	26.7	0.2	10	19.3	36.6	89.1
Manual consensus	28.8	0.5	12.1	21.2	39.8	90.5
VDS‐based DIA	39.6	0.7	19.4	33.3	54.5	98.8
DL‐based DIA	38.3	0.7	18.0	31.6	52.0	95.4

DIA, digital image analysis; DL, deep learning; VDS, virtual dual staining.

### Comparison between manual scoring and VDS‐based DIA

**Figure 4:**
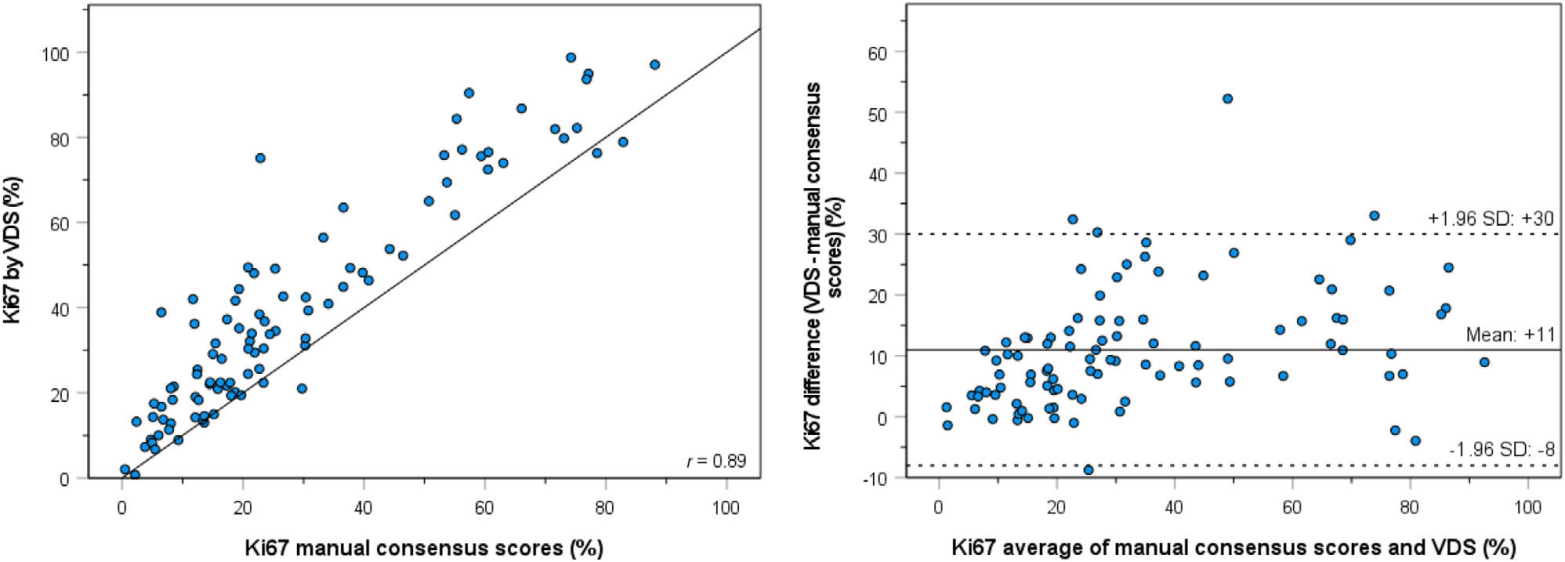
Scatterplot with the correlation coefficient (*r*) (left) and Bland–Altman plot of agreement (right) between Ki67 hotspot PIs by manual scoring versus automated hotspot detection and analysis by the VDS‐based algorithm. [Color figure can be viewed at wileyonlinelibrary.com]

Ki67 hotspot PIs obtained with the VDS‐based algorithm are shown in Table 2. Ki67 hotspot PIs obtained by automated hotspot detection and scoring by the VDS‐based algorithm were higher than the manual consensus scores, with a mean of 39.6% versus 28.8% and a median of 33.3% versus 21.2%. Scatterplots and Bland–Altman plots of automated hotspot detection and scoring by the VDS‐based algorithm compared to manual counting are shown in Figure 4.The intermodality agreement between manual counting and the automated hotspot analysis was substantial (*r* of 0.90).

### Comparison between manual scoring and DL‐based DIA

**Figure 5:**
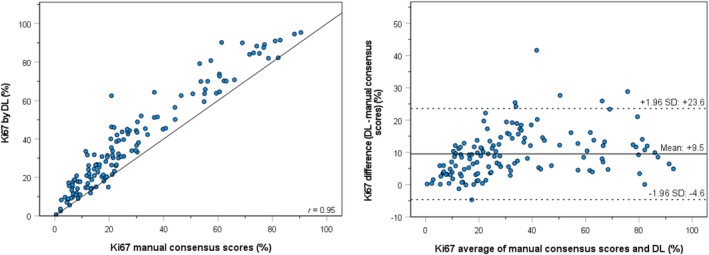
Scatterplot with the correlation coefficient (*r*) (left) and Bland–Altman plot of agreement (right) between Ki67 hotspot PIs by manual scoring versus automated hotspot detection and analysis by the DL‐based algorithm. [Color figure can be viewed at wileyonlinelibrary.com]

Ki67 hotspot PIs obtained with the DL‐based algorithm are shown in Table 2. Ki67 hotspot PIs by automated hotspot detection and scoring by the DL‐based algorithm were higher than the manual consensus scores, with a mean of 38.3% versus 28.8% and a median of 31.6% versus 21.2%, respectively. Scatterplots and Bland–Altman plots of automated hotspot detection and scoring by the DL‐based algorithm compared to manual counting are shown in Figure 5. Agreement between manual assessment and DL‐based DIA was high (*r* of 0.95).

### Cases with >1.96 SD Ki67 difference

**Figure 6:**
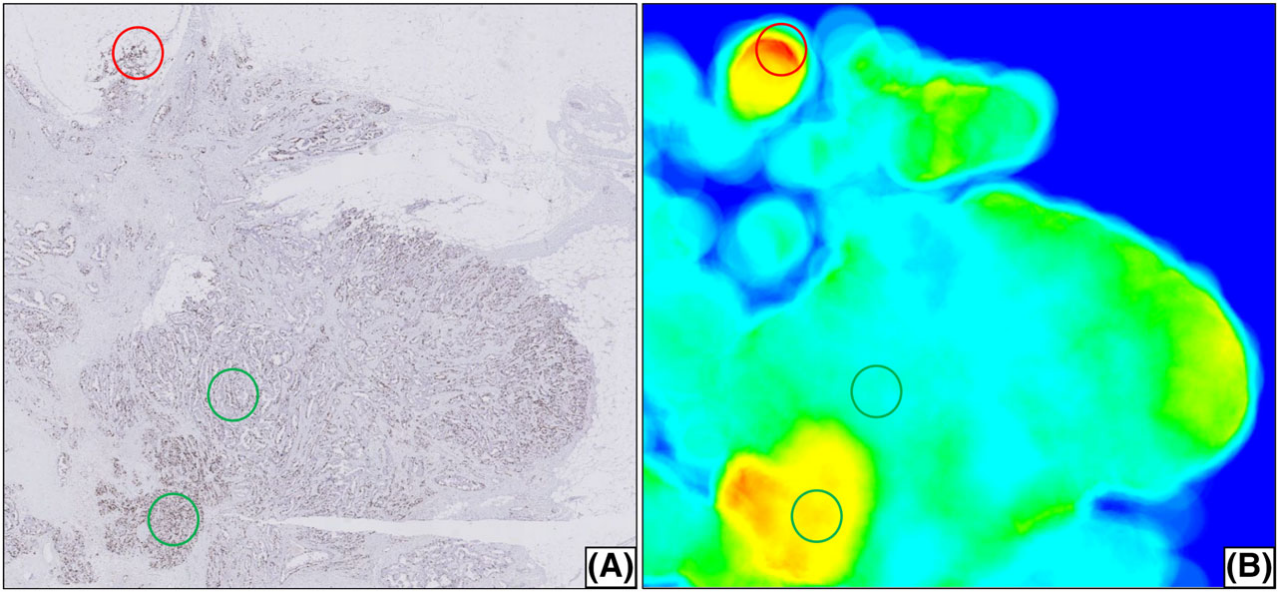
Ki67‐stained slide (**A**) and the corresponding heatmap created by the DL‐based algorithm (**B**), with the manually annotated hotspots (green) and the hotspot detected by the algorithm (red). [Color figure can be viewed at wileyonlinelibrary.com]

Five out of 98 cases (5.1%) showed a difference of >1.96 SD between the Ki67 PI obtained by VDS‐based DIA and manual scoring (Figure 4). For DIA, 7 out of 135 cases (5.2%) showed a difference of >1.96 SD. In these cases, the DIA methods located the hotspot at the invasive margin of the tumour, containing an area with almost exactly 500 tumour cells. The manual observers tended to locate a hotspot within the tumour and annotated an area with a high density of tumour cells. A representative example is shown in Figure 6.

## Discussion

Although the Ki67 PI has been widely used as a prognostic and predictive marker in breast cancer, due to the lack of standardized and reproducible counting methods it remains unclear what method, global or hotspot scoring, is the most clinically relevant. In this study we aimed to assess the performance of two automated Ki67 proliferation hotspot detection and scoring algorithms for invasive breast carcinoma. We found that automated hotspot detection and analysis based on VDS and DL on whole slides correlated strongly with manual assessment and led to higher Ki67 hotspot PIs compared to manual assessment.

The International Ki67 in Breast Cancer Working Group (IKWG) has performed a series of studies that showed that a high level of concordance can be achieved using clear scoring instructions for visual Ki67 assessment, although the intraclass correlation coefficient (ICC) for global scoring was higher than for hotspot scoring.^22^ Similarly, we found a high correlation between the two manual Ki67 hotspot PIs (*r* = 0.87).

In our previous study validating a global Ki67 scoring algorithm, we showed that DIA is an accurate and reproducible alternative to manual scoring in predefined ROIs.^12^ Several other studies used automated Ki67 hotspot detection and scoring algorithms based on the VDS or Virtual Triple Staining method and showed increased reproducibility and higher Ki67 hotspot PIs compared to manual scoring.^11^, ^16^, ^24^, ^25^ However, to our knowledge, we are the first to use a DL‐based algorithm for automated Ki67 hotspot detection and scoring in breast cancer. This has the advantage that it is not dependant on the virtual alignment of the CK8/18 and Ki67 stained slides. In our current study, a substantial number of 37 cases (27%) were excluded due to failure of VDS. A physical double stain could have been used to overcome alignment issues leading to failure of VDS, but different stains within one slide may show ‘bleeding’ of staining, which compromises assessment.^26^, ^27^ DL‐based tumour cell detection on immunohistochemistry slides circumvents the need of dual staining, thereby leading to less or no exclusion of cases. Furthermore, the DL‐based algorithm correlated stronger with manual scores than the VDS‐based algorithm, offering a more accurate alternative to manual scoring. Therefore, the DL‐based algorithm outperforms both manual scoring and the VDS‐based algorithm in clinical applicability.

The IKWG has developed guidelines aiming to reduce the variation and to improve comparison of Ki67 analysis.^8^ The methods of scoring Ki67 in breast cancer studies vary significantly, with approaches ranging from focussing solely on Ki67 hotspots to complete avoidance of hotspots. Until the most clinically relevant scoring method is clarified, the guidelines of the IKWG recommend whole‐slide assessment of Ki67 immunohistochemistry, and to include hotspots in the overall average score. However, in recent years multiple studies have shown prognostic potential of Ki67 hotspots in breast cancer in terms of disease‐free survival and overall survival.^11^, ^18^, ^19^, ^20^, ^21^, ^25^, ^28^, ^29^ Aleskandarany *et al*. found a perfect correlation between the Ki67 hotspot PI and Ki67 expression in lymph node metastasis, indicating that the Ki67 hotspot represents the part of the primary tumour that is most likely to metastasize.^29^ This further suggests the potential clinical relevance of Ki67 hotspots.

Several studies have shown that DIA not only offers standardized and reproducible assessment of Ki67 in breast cancer, but also significantly contributes to its prognostic value.^11^, ^30^, ^31^, ^32^ However, there is still discussion in which region of the tumour the Ki67 PI should be assessed. This discussion is partly hampered by the lack of robust assessment methods for tumour subregions.^11^ Furthermore, consensus is needed on the number of tumour cells that should be counted, since this can affect Ki67 PIs.^28^ Automated Ki67 hotspot analysis using DL may further aid in predicting disease outcome and might be used in prospective studies further investigating the prognostic and predictive value of the Ki67 PI in tumour subregions and determine the optimal number of tumour cells to be counted. In addition, the use of DL‐based DIA extends beyond Ki67 hotspot detection and scoring. DL‐based DIA could also aid in standardizing global Ki67 scoring.^10^ Furthermore, the heatmap created by the algorithm can reveal the heterogeneity of Ki67 in tumour samples, which may be of clinical significance. Laurinavicius *et al*. demonstrated that spatial heterogeneity can serve as an independent prognostic indicator of overall survival in breast cancer patients.^33^

Due to the retrospective nature of the study, the heterogeneity of the study group and the lack of follow‐up data no statements can be made about the prognostic and predictive power of the different automated Ki67 PI assessment methods, which are limitations of this study.

In conclusion, automated Ki67 hotspot detection and analysis correlated strongly with manual Ki67 assessment. Automated Ki67 hotspot assessment led to more accurate hotspot detection and higher proliferation indices compared to manual scoring. The DL‐based algorithm outperformed the VDS‐based algorithm in clinical applicability, because it does not depend on virtual alignment of slides. Furthermore, the DL‐based method correlated stronger with manual scores. Use of a DL‐based algorithm may allow clearer Ki67 PI cutoff values, thereby improving the clinical usability of Ki67. Although several studies have demonstrated the predictive value of the Ki67 hotspot PI in breast cancer patient outcome, integration in clinical decision making remains limited. Future clinical studies should further investigate the clinical significance of DL‐based Ki67 hotspot assessment in breast cancer patients.

## Conflict of interest

BvdV reports honoraria received by UMCG for expertise or scientific advisory board/consultancy (on request): Visiopharm, Philips, MSD/Merck, Daiichi‐Sankyo/AstraZenica; Speaker's fee from Visiopharm, Diaceutics, MSD/Merck. Unrestricted grants from Owkin and GE Healthcare. Personal fees from DEKRA. All unrelated to the current article. The other authors declare no conflicts of interest.

## Patient consent statement

This retrospective study was deemed to fall outside the scope of the Medical Research Involving Human Subjects Act (WMO) by the Ethical Committee of the UMCG (ValiDIApath, research register number 16818, UMCG research register number 18848); therefore, informed consent was not required according to the Dutch Law for Medical Research and institutional guidelines.

## Supporting information


**Table S1.** Comparison of means of manual scoring, VDS‐based DIA and DL‐based DIA.
**Table S2.** Agreement between manual scoring, VDS‐based DIA and DL‐based DIA.

## Data Availability

The datasets generated and/or analysed during the current study are available from the corresponding author on reasonable request.
