# Supplementary material for: Advancing Ki67 hotspot detection in breast cancer: a comparative analysis of automated digital image analysis algorithms
Source: Histopathology. 2024 Aug 5;86(2):204–13. doi: 10.1111/his.15294 (PMC11649514; doi:10.1111/his.15294)
Supplement: Supplementary file 1 — Table S1. Comparison of means of manual scoring, VDS‐based DIA and DL‐based DIA. Table S2. Agreement between manual scoring, VDS‐based DIA and DL‐based DIA. [file HIS-86-204-s001.docx]

**Supplementary data**

**Supplementary Table 1.**

|  |  |  | 95% Confidence Interval of the Difference | |  |  |  |  |
| --- | --- | --- | --- | --- | --- | --- | --- | --- |
|  | **Mean** | **Std Deviation** | **Std Error Mean** | **Lower** | **Upper** | **t** | **df** | **Sig (two-tailed)** |
| Manual consensus – VDS-based DIA | -10.95 | 9.70 | 0.98 | -12.90 | -9.01 | -11.17 | 97 | <0.001* |
| Manual consensus – DL-based DIA | -9.53 | 7.16 | 0.62 | -10.75 | -8.31 | -15.47 | 134 | <0.001* |
| VDS-based DIA – AI-based DIA | 1.78 | 7.46 | 0.75 | 0.29 | 3.28 | 2.36 | 97 | 0.020* |

*P-value ≤ 0.05

**Supplementary Table 2. Agreement between manual scoring, VDS-based DIA and DL-based DIA**

|  | **VDS-based DIA** | **DL-based DIA** |
| --- | --- | --- |
| **Manual observer 1** | 0.89 | 0.94 |
| **Manual observer 2** | 0.85 | 0.89 |

*DIA* digital image analysis, *VDS* virtual dual staining, *DL* deep learning
